# Duplex Kidney Anomalies and Associated Pathologies in Children: A Single-Center Retrospective Review

**DOI:** 10.7759/cureus.25777

**Published:** 2022-06-09

**Authors:** Sevim Yener, Cemile Pehlivanoğlu, Zeliha Akis Yıldız, Huri T Ilce, Zekeriya Ilce

**Affiliations:** 1 Department of Pediatric Urology, University of Health Sciences Umraniye Training and Research Hospital, Istanbul, TUR; 2 Department of Pediatric Nephrology, University of Health Sciences Umraniye Training and Research Hospital, Istanbul, TUR; 3 Department of Pediatric Surgery, University of Health Sciences Umraniye Training and Research Hospital, Istanbul, TUR; 4 Department of Nuclear Medicine, Sakarya University Faculty of Medicine Training and Research Hospital, Sakarya, TUR; 5 Department of Pediatric Surgery, University of Health Sciences, Umraniye Training and Research Hospital, Istanbul, TUR

**Keywords:** ureterocele, hydronephrosis, bifid pelvis, ureteral duplication, dupleks kidney

## Abstract

Introduction: Duplex renal collecting systems are one of the most common congenital anomalies of the urinary tract. The exact prevalence of this anomaly is difficult to ascertain because most patients are asymptomatic, and the abnormality is frequently detected incidentally. The aim of this study is to retrospectively review the demographic characteristics and different clinical presentations, related pathology, and treatment methods of patients with duplex system anomaly who applied to our institution, with a literature review.

Methods: This is a retrospective study, performed at the Department of Pediatric Urology and Pediatric Surgery, Umraniye Training and Research Hospital, a tertiary center, from 2010 to 2021. Age, gender, presenting symptoms, and associated anomalies were determined in all patients. Asymptomatic patients with variants of duplex kidney anomaly detected incidentally did not require any surgical intervention. Necessary surgical interventions were performed depending on the pathologies of other symptomatic patients associated with duplex kidney anomaly variants.

Results: A total of 239 patients had duplex systems. The patients were divided into two groups according to their age, 0-24 months (newborn and infant) and over 24 months. There were 45 (18.8%) patients in the 1st group and 194 (81.1%) patients in the 2nd group. It was seen that the most common symptom in 85 (35.6%) patients was urinary tract infection (UTI). It was observed that 5 (2%) patients had no symptoms and were detected during routine screening. When comorbidities detected with the duplex system were examined, the most common ones were antenatal hydronephrosis 23 (9.6%). Ureterocele excision was performed in ten patients, laparoscopic heminephrectomy was performed in six patients, and ureteroneocystostomy was performed in one patient.

Conclusions: It is important that magnetic resonance urography (MRU) duplex renal collecting systems, which is a current imaging method used in the evaluation of the duplex system, provide detailed information about the morphology and function and are useful in the evaluation of associated anomalies. Diagnosis and treatment before it becomes symptomatic or results in further kidney damage are important for the preservation of renal function in advanced follow-ups.

## Introduction

A duplex kidney is defined as a kidney unit consisting of two pelvicalyceal systems and is one of the common congenital anomalies of the urinary tract [1-3]. Overall, ureteral duplication was reported in one in 125 (0.8%) cases in the autopsy series [2-4]. Hartman and Hodson reported a higher incidence of 2%- 4% in a clinical series of patients with urinary symptoms [5]. In a large study of 700 children presenting with urinary tract infection, ureteral duplication was detected in 8% of patients [6]. Right and left kidneys are equally affected in duplex system anomalies. The bilateral duplex system is seen in approximately 20% to 40% of affected individuals [3,6,7]. It is reported that female patients are affected two times more than male patients [6-8]. Most duplex abnormalities are asymptomatic [9]. Duplex kidneys appear symptomatically only when associated with complications and associated anomalies. Although there are efforts to determine the incidence with autopsy studies in these patients, the number of cases is limited. Studies are mostly about the procedures and results in patients who have clinical findings and have undergone surgery. In the literature, studies on cases without clinical findings are quite limited.

The aim of this study is to retrospectively review the demographic characteristics and different clinical presentations, related pathology, and treatment methods of patients with duplex system anomaly who applied to our institution, with a literature review.

## Materials and methods

Duplex system patients who applied to Umraniye Training and Research Hospital pediatric urology, pediatric surgery, pediatric nephrology, and all other pediatric clinics between 2010-2021 were retrospectively analyzed. Ethics committee approval was obtained for the study (approval number B.10.1.TKH.4.34.H.GP.0.01/293).

Patients who applied for different symptoms and were found to have a duplex system were included in the study. During this period, a duplex system was detected in a total of 239 patients. Age, gender, presentation, and associated anomalies were determined in all patients. The results of imaging methods performed on these patients were recorded. These methods vary according to the patient; urinary system ultrasonography, VCUG (voiding cystourethrography), DMSA (dimercaptosuccinic acid), and MAG-3 (mercaptoacetyltriglycine) renal scintigraphy, MRU (magnetic resonance urography) images were recorded. Those with antenatal findings were followed up and treated according to current guidelines. The surgical procedures performed were determined. Asymptomatic patients with variants of duplex kidney anomaly detected incidentally did not require any surgical intervention. Necessary surgical interventions were performed depending on the pathologies of other symptomatic patients associated with duplex kidney anomaly variants. Cold knife, bugbee and holmium laser were used for endoscopic ureterocele incision. Vesicoureteral reflux was detected in 13 patients. Bilateral vesicourethral reflux was detected in two patients (bilateral both poles), left (5 in the upper pole and 3 in the lower pole) in eight patients, and right (upper pole) in three patients. One of the patients with reflux on the left side was after a ureterocele excision. Vesicoureteral reflux (VUR) treatment was performed using dextranomer/hyaluronic acid with the STING method. All patients who underwent cystoscopy underwent retrograde pyelography (RGPG) for diagnostic purposes. The laparoscopic method was preferred for patients who required partial nephrectomy. Partial nephrectomy indication; non-functioning upper pole and recurrent urinary tract infection. For patients requiring ureterocele excision and reimplantation, a trans trigonal ureteroneocystostomy procedure was performed. Since our study did not require any statistical calculations or comparisons between groups, no special statistical study was required.

## Results

A total of 239 patients had duplex systems, 143 female (60%) and 96 male (40%). The patients were divided into two groups according to their age, 0-24 months (newborn and infant) and over 24 months. There were 45 (18.8%) patients in the 1st group and 194 (81.1%) patients in the 2nd group.

Patients were also examined according to the sides of duplex renal collecting system anomalies. The right duplex system was detected in 133 patients (55.6%), and the left duplex system was detected in 84 patients (35.1%). In 22 patients (9.2%), a bilateral duplex system was observed.

When evaluated according to the presenting symptoms and comorbidities, it was seen that the most common symptom in 85 (35.6%) patients was urinary tract infection (UTI). In order of frequency, abdominal pain 34 (14.2%), urinary incontinence (27) (11.3%), dysuria 10 (4.2%), hematuria 9 (3.7%), jaundice 5 (2%) , fever in 5 (2%), vomiting in 2 (0.8%), diarrhea in 1 (0.4%), growth retardation in 1 (0.4%) and trauma in 1 (0.4%) patient. It was observed that 5 (2%) patients had no symptoms and were detected during routine screening (Table 1).

**Table 1:** Patients' age groups and clinical presentations

	Age_Group					
	0-24 months			24 months and older		
	n		%	n	%	
UTI		15	17,6%	70	82,4%	
Abdominal pain		0	0,0%	34	100,0%	
İncontinence		0	0,0%	27	100,0%	
Dysuria		1	10,0%	9	90,0%	
Hematuria		0	0,0%	9	100,0%	
No Symptoms		1	20%	4	80%	
Fever		3	60,0%	2	40,0%	
Jaundice		5	100,0%	0	0,0%	
Trauma		0	0,0%	2	100,0%	
Vomiting		1	50,0%	1	50,0%	
Diarrhea		0	0,0%	1	100,0%	

**Figure 1:**
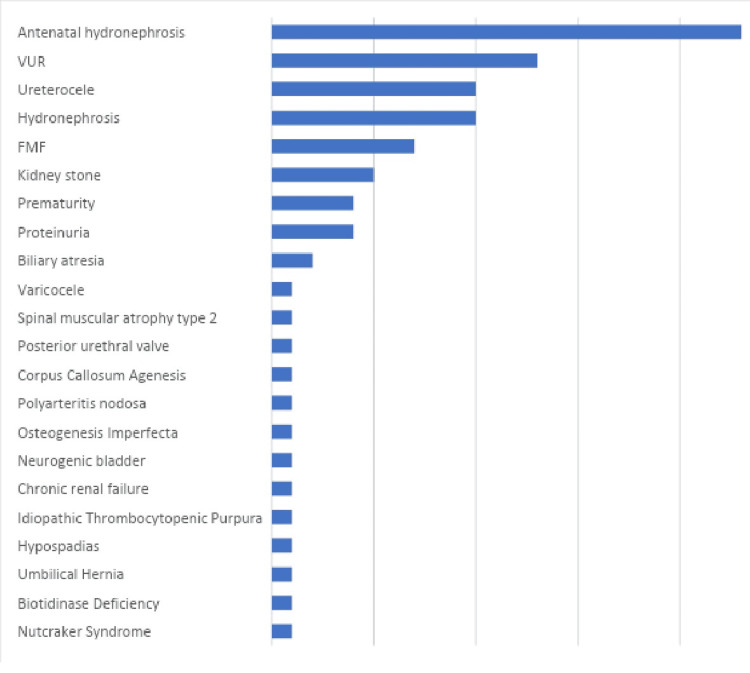
Comorbidities associated with the duplex renal system

When comorbidities detected with the duplex system were examined, the most common ones were antenatal hydronephrosis 23 (9.6%), vesicoureteral reflux 13 (5.4%), ureterocele 10 (4.2%) and hydronephrosis (postnatal) 10 (4.2%) patients (Figure 1). 

Since all patients were scanned for the urinary tract ultrasonography all patients were included in the urinary system USG group. Forty-three patients in VCUG, 127 patients in DMSA, 26 patients in MAG-3, and eight patients in the MRU group. Since the diagnosis of the duplex system is determined by USG, all patients underwent the urinary system USG scan. Patients with recurrent urinary tract infections and hydronephrosis detected on urinary USG were evaluated with VCUG and DMSA. Patients with hydronephrosis and suspected obstruction were evaluated with MAG-3 scintigraphy. In selected patients with the duplex system, it was evaluated with MRU in order to clearly reveal the anatomy.

Thirteen of the patients had vesicoureteral reflux. Bilateral vesicourethral reflux was detected in two patients (bilateral both poles), left (5 in the upper pole and 3 in the lower pole) in eight patients, and right (upper pole) in three patients. Bilateral grade 3 reflux was found in one of the patients with bilateral reflux, and grade 3 on the left and grade 2 on the right in the other. Grade 3 reflux was detected in five of eight patients on the left side, grade 4 in one patient, and grade 2 reflux in two patients. Grade 3 reflux was found in one patient with reflux on the right side, and grade 2 reflux was found in the other two patients. The patients were followed up under prophylaxis with monthly urine culture and urinary USG after injection. If there was no urinary tract infection in the follow-up, VCUG was performed at the postoperative sixth month time. DMSA was detected to leave a scar in 39 of the 127 patients taken. MAG-3 has been observed obstructive in four of the 26 patients withdrawn.

Ureterocele excision was performed in ten patients, laparoscopic heminephrectomy was performed in six patients, and ureteroneocystostomy was performed in one patient. Diagnostic cystoscopy and retrograde pyelography were performed on 29 patients with the suspected duplex system, hydronephrosis, urinary tract infection, and ureterocele on urinary USG. Ureterocele of patients with ureterocele was incised. After the incision, monthly urine culture and USG follow-up were performed under follow-up prophylaxis. Partial nephrectomy indication; non-functioning upper pole and recurrent urinary tract infection. One patient had a recurrent urinary tract infection and a very large ureterocele closing the urethral outlet. As he had reflux at the same time, ureterocele excision and ureteroneocystostomy were performed Tables 2-3). 

**Table 2:** Patients' age groups and surgical interventions

		Age_Group						
		0-24 months			24 months and older			
		n		%	n		%	
Ureterocele excision		7	70.0%		3	30.0%		
Partial nephrectomy		4	57,1%		2	33,3%		
Ureteroneocystostomy		1	100,0%		0	0,0%		
Subureteric injection		0	0,0%		4	100%		

**Table 3:** Clinical presentation of patients undergoing surgery

		Surgical intervention			
		Yes		No	
		n	%	n	%
UTI		6	7,06%	79	92,94%
Abdominal pain		2	5,88%	32	94,12%
İncontinence		0	0,00%	27	100,00%
Dysuria		0	0,00%	10	100,00%
Hematuria		1	11,11%	8	88,89%
No Symptoms		0	0,00%	6	100,00%
Fever		1	20,00%	4	80,00%
Jaundice		0	0,00%	5	100,00%
Trauma		0	0,00%	2	100,00%
Vomiting		0	0,00%	2	100,00%
Diarrhea		0	0,00%	1	100,00%

## Discussion

The duplex renal collecting system is one of the most common congenital anomalies of the urinary system [1]. Multiple classification systems have been proposed to categorize this pathology, as duplex systems can appear in a variety of ways. The naming and classification of duplex systems are done as specified by the Terminology Committee of the Urology Division of the American Academy of Pediatrics [10]. In addition to this standard nomenclature, in the rare case of reverse Y-ureteral duplication, the two ureters merge before entering the kidney [11]. Another blind ureter is defined as the ureter that ends without draining into the bladder. A very rare H-shaped ureter has also been reported. Although the majority of cases involve a simple duplex system, up to six independent multiple ureters have been described. Ureteroceles are among the various anomalies associated with the duplex system [12,13].

There are many variations within this situation. A complete duplex kidney, consisting of two ureteric buds originating from the mesonephric duct, has two separate pelvicalyceal systems and two ureters. These ureters fuse separately with the developing renal blastoma, resulting in two separate excretory systems of the kidney [14]. The ureters are referred to as the upper and lower pole ureter, depending on the draining renal segment. The upper pole ureter is characteristically located more medially and caudally than the cranially and lateral opening of the lower part. This relationship is called the Weigert-Meyer rule in more than 90% of cases. In a partial duplication anomaly, there is single ureter budding, resulting in a premature division before encountering the mesenchyme [11,12].

In the literature, it is mentioned that the frequency of duplex kidney variants is predominant in girls [8,9,11]. In our series, 143 cases were female (60%), and 96 were male (40%). It is reported that right and left kidneys are equally affected in duplex system anomalies. Bilateral incidence is seen in approximately 20% to 40% of patients with duplex renal systems [15,16]. The female to male ratio of the patients in our series is also predominant as in the literature. However, when the patients were evaluated for sides, it was determined that the patients were predominantly affected in the right system (55%). On the other hand, contrary to the literature, bilateral involvement was found to be lower (9%). While the patients in the other series constituted the group of patients who underwent surgery due to the duplex renal system [17,18], our series consists of a wide spectrum, including the patients with duplex renal systems in ultrasonography, with or without the surgical procedure.

In the planning of the study; patients with prenatal diagnosis, early clinical findings, and late clinical findings were divided into two groups. In our study, two patients were identified in the first group consisting of 0-24 months old patients. It was observed that these patients were diagnosed with the antenatal duplex renal system. The majority of the patients, which is the second group (n: 194), constitute the age group over 24 months.

When the presenting symptoms were evaluated according to age groups, urinary tract infection was found to be the most common symptom in the 1st (n:70) and 2nd groups (n:14), similarly. The second presentation symptom was fever in the 1st group (n:3), and urinary incontinence in the 2nd group (n:27). It was determined that five patients were diagnosed during routine controls without any symptoms. In the study by Okay et al., urinary tract infection, antenatal hydronephrosis, and pain were the most common presenting symptoms in the series of 13 patients with a duplex renal system diagnosis and an average age of 5 years, who underwent a surgical procedure. It has been reported that no clinical findings were found in cases with a postnatal diagnosis with antenatal suspicion [19]. Luo et al. reported the most common urinary tract infections (46%), incontinence (20%), and antenatal or postnatal diagnosis (18%) [20]. The patients included in the study were divided into three groups: 0-1 years old, 2-3 years old, and over 4 years old. Almost the same number of patients were found in the first and second groups. Similar to our study, urinary tract infection is the most common presenting symptom in the advanced age group.

In this context, the risk of kidney infection in children increases 20 times with increasing age. Many of these patients require some form of medical or surgical intervention in their childhood or early adolescence [21,22], with recurrent urinary tract infections, urinary incontinence due to ectopic ureteral opening, or impaired renal function [23]. Therefore, early diagnosis is important. The fact that the second group had the highest number of patients in our series and the urinary tract infections was the most common symptoms in our series supports the literature.

The second most common symptom detected with the duplex renal system was urinary incontinence. Routine USG scanning is thought to be helpful in detecting renal anomalies in patients presenting with urinary incontinence. However, our patients in this group did not have a history of urinary incontinence suggesting ectopic opening when they applied to the clinic. Since most of the patients had a history of urinary incontinence only at night, no further examination was performed for ectopic opening. No pathology that required surgery or caused urinary tract infection was detected in this group of patients. In fact, these patients were non-symptomatic patients who would not have had a duplex system if they had not been screened for incontinence.

The upper pole ureter may open into the prostatic urethra in boys and into the urethra or vagina in girls. In our series, ureterocele was detected in only 10 patients. In two female patients with complete duplication and ureterocele, ectopic ureteral orifice was opening into the urethra in one and the bladder neck in the other. In a male patient, the ectopic ureter was opening to the bladder neck. Incontinence of these patients could not be evaluated since they are young age and were not toilet trained yet.

In the English literature we reviewed, there is almost no study evaluating the diseases associated with the duplex system. The most common associated diseases in our series were antenatal hydronephrosis (n:23%, 9.6%) and vesicoureteral reflux (n:13, 5.4%). Most of the associated diseases were urinary system diseases (n:71 29.7%). However, it was observed that diseases other than the urinary system were also associated, albeit in a small number (n:20,8.3%).

The use of dimercaptosuccinic acid scintigraphy to define kidney function was demonstrated in a study by Kullendorff and Wallin. Renal function was severely impaired in 44% of cases with duplex kidneys [24]. However, these patients are symptomatic, even those requiring surgery. In our series, scars were detected in 39 of 127 patients who underwent DMSA.

Patients who had recurrent urinary tract infections and did not have additional anatomical urinary anomalies were evaluated using the top-down approach, and DMSA was withdrawn beforehand. Voiding cystourethrography was performed to evaluate the patients with scars in terms of vesicoureteral reflux. Vesicourethral reflux was detected in 13 (5.4%) patients. The injection was performed in four patients diagnosed with reflux. Ureteroneocystostomy and ureterocele excision was performed on one patient. The reflux of the remaining patients was observed to regress in the follow-up under prophylaxis.

There are studies reporting the effectiveness of MRU in revealing the anatomy of duplex system patients [25,26]. In the study, this imaging method was used for a small group of eight patients. The necessity of anesthesia for this examination in young children has brought about the preference for selected patients.

When serial scans are examined, there are various studies that include surgical procedure comparisons. There are no large series of these cases in the pediatric patient group. Therefore, sufficient information about the treatment plan is not available. Another reason why standard procedures could not be established in treatment protocols is the variety of anomalies. It is stated that no single method is superior as a definitive treatment, but the management of each duplex kidney should be planned individually for the patient.

In our series, when the patients who underwent surgery were evaluated according to age groups, it was seen that partial nephrectomy was performed on four patients in the 0-24 months age group, and two of the three patients in the group over 24 months old. It was determined that one patient who underwent trans trigonal ureteroneocystostomy was included in group 2 patients.

As a result, while endoscopic ureterocele excision and partial nephrectomy were the surgical procedures performed more frequently in our first group of patients, it was seen that the second group was more likely to have injections due to vesicoureteral reflux. We think that early recognition of these patients is important, as they can be effectively treated with early intervention in complicated cases and when symptomatic.

## Conclusions

In conclusion, the duplex renal collecting system is one of the most common congenital anomalies of the urinary system. The diagnosis rate is increasing with the frequent use of urinary system ultrasonography in the screening of prenatal and postnatal urological diseases.

It is important to evaluate patients with the duplex system in prenatal or postnatal urinary USG in the pediatric age group in terms of urinary tract infection with a complete urinalysis and urine culture. In the absence of hydronephrosis and urinary tract infection, clinical follow-up with routine USG and urine culture is sufficient. If an obstructive urinary system pathology is considered in the presence of hydronephrosis, it is recommended to be evaluated with MAG-3 scintigraphy. In the presence of hydronephrosis, vesicoureteral reflux investigation is performed with VCUG and a filling defect of the ureterocele can be detected. It is recommended to evaluate the presence of scarring in the kidney and detailed upper-lower pole renal function with DMSA scintigraphy. In patients with ureterocele, diagnostic cystoscopy is performed to evaluate the ureterocele structure and ectopic orifice. If necessary, intervention to vesicoureteral reflux can be done endoscopically. We recommend RGPG to all patients undergoing cystoscopy in order to clarify the anatomy of the duplex system. It is reported that MRU, which is another current imaging method used in the evaluation of the duplex system, provides detailed information about the morphology and function of the duplex renal collecting systems and is useful in the evaluation of associated anomalies. However, since it requires anesthesia for image quality in younger age group patients, it is appropriate for only selected cases.

In addition, in older female patients, the symptom of persistent urinary incontinence should be a warning for ectopic orifice opening. At the same time, recurrent episodes of epididymorchitis should also be a warning for ectopic orifice opening in male patients.

The surgical method to be chosen in duplex system cases differs in each patient according to the type and degree of the anomaly. In addition to medical and conservative treatment in low-grade VUR and ureteroceles, follow-up and treatment success with endoscopic interventions has been reported positively. Laparoscopic partial nephrectomy is a suitable, safe and effective option for dysfunctional upper pole kidneys and its results are similar to open surgery. In patients with giant ureterocele, bladder outlet obstruction, and high-grade reflux, ureterocele excision and ureteroneocystostomy are good surgical options. Diagnosis and treatment before it becomes symptomatic or results in further kidney damage are important for the preservation of renal function in advanced follow-ups.
